# Structurally distinct cyclosporin and sanglifehrin analogs CRV431 and NV556 suppress established HCV infection in humanized-liver mice

**DOI:** 10.1371/journal.pone.0237236

**Published:** 2020-08-07

**Authors:** Michael Bobardt, Magnus Joakim Hansson, Patrick Mayo, Daren Ure, Robert Foster, Philippe Gallay

**Affiliations:** 1 Department of Immunology & Microbiology, The Scripps Research Institute, La Jolla, California, United States of America; 2 NeuroVive Pharmaceutical AB, Lund, Sweden; 3 Hepion Pharmaceuticals, Edison, New Jersey, United States of America; Inserm U0152, UMR 5286, FRANCE

## Abstract

We previously reported that the non-immunosuppressive cyclophilin inhibitors (CypIs)—cyclosporin A analog CRV431 and sanglifehrin analog NV556—efficiently inhibit HCV replication *in vitro*. In this study, we asked whether they can also reduce HCV replication *in vivo*. We found that a single oral administration of CRV431 and NV556 to HCV-infected humanized-liver mice drastically reduced HCV blood levels. The antiviral effect was observed when CRV431 or NV556 were each individually administered with HCV, 3, 6 weeks or even 3 months post-infection when viral replication is robust. These results were confirmed in chimeric mice implanted with human hepatocytes isolated from three distinct donors. Remarkably, no viral rebound was observed 5 months after a single dose administration of 50 mg/kg of CRV431 or NV556 four weeks post-HCV infection, indicating the possibility of suppression of an established viral infection. Since we recently demonstrated that both CRV431 and NV556 also inhibit the development of liver fibrosis and hepatocellular carcinoma in nonviral-induced non-alcoholic steatohepatitis mouse models, our present data suggest that the two entirely structurally different CypIs—CRV431 and NV556—derived from unrelated natural products, represent attractive partners to current direct-acting agent (DAA) regimens for the treatment of hepatitis C and liver diseases.

## Introduction

Despite recent therapeutic progress, HCV remains a worldwide health issue with 300,000 deaths per year. HCV-infected patients develop cirrhosis (27%) and HCC (25%) [1–3]. Earlier than 2011, the standard treatment for HCV infection was the combination of pegylated interferon alpha (pIFNa) and ribavirin (RBV) for 24 or 48 weeks contingent on the HCV genotype [4], resulting in a sustained virologic response (SVR) rate of 40–50%, but associated with major side effects [5–7]. The first two direct-acting antivirals (DAAs)–the protease inhibitors telaprevir and boceprevir–enhanced viral clearance rate by 70% compared to the pIFNa and RBV treatment, but still induced significant side effects [8–9]. Then, new FDA-approved DAAs were developed, including the protease inhibitors glecaprevir, grazoprevir and simeprevir, the NS5B polymerase inhibitor sofosbuvir and the NS5A inhibitors daclatasvir and elbasvir [10].

DAA resistance, which is associated with the selection of resistance-associated substitutions (RASs) present at baseline or are acquired during treatment [11], remains a main issue for the treatment of HCV patients [11]. Given the high number of patients who will need retreatment, an option to circumvent DAA resistance is to include into DAA regimens drugs with high barrier to resistance and with distinct antiviral mechanisms of action. Such a class of candidates are the cyclophilin inhibitors (CypIs). We and others showed that CypIs inhibit the replication of diverse viruses including HIV-1, HCV, HBV, arteriviruses and coronaviruses [12–15]. Their most striking inhibitory effect was demonstrated for HCV [16–22]. Specifically, the CypI alisporivir/Debio-025 exhibited high antiviral potency *in vitro* as well as in HCV-infected patients in phase I, II, and III studies [16–22]. There are two structurally distinct main classes of CypIs, which are derived from two natural products–cyclosporin A and sanglifehrin A: i) the non-immunosuppressive cyclosporine A (CsA) analogs such as CRV431 (previously named CPI-431-32) [13]; and ii) the non-immunosuppressive sangliferin analogs such as NV556 (previously named BC556/NVP018) [23]. Both classes of CypIs neutralize the peptidyl-prolyl *cis-trans* isomerase (“foldase”) activity of members of the cyclophilin family by binding to their enzymatic hydrophobic pockets [13, 23]. Both classes of CypIs show efficacy against HIV-1 and HCV [13, 23] because they block the formation of complexes between cyclophilins–especially the abundant cytosolic cyclophilin A (CypA)—and the respective viral ligands, HIV-1 capsid [24–26] and HCV NS5A [27–30]. It has been postulated that the inhibitors disrupt the proper folding of HIV-1 capsid and HCV NS5A and in turn the optimal progression of the viruses through their life cycles and productive infection of CD4+ cells and hepatocytes, respectively.

Although we previously showed and reported that the non-immunosuppressive cyclosporin A and sanglifehrin A or B analogs derived from distinct natural products with highly dissimilar structures–CRV431 and NV556 –block HCV replication *in vitro* [13, 23], we asked in the present study whether they could also suppress HCV infection *in vivo* in humanized-liver chimeric mice.

## Materials and methods

### Drugs

CRV431 was synthesized in-house by chemical modification of cyclosporin A while NV556 was manufactured by a three-step semi-synthesis from a fermentation product. Their purity exceeded 95% as determined by HPLC. Sofosbuvir was purchased from MedChemExpress.

### Animal care

Animal housing: individually ventilated cage (IVC) racks are used to house the majority of mice. HEPA-filtered air is supplied into each cage at a rate of 60 air changes per hour. Mice are housed in solid bottom cages. Static mouse cages are changed at least once a week. IVCs are changed at least once every14 days. Certain strains of rodents (e.g., diabetic) are changed into clean cages more frequently as needed. Room environment: heating, ventilation and air conditioning performance is routinely assessed as part of facility renovations, system repairs, and at least once every 3 years. Each animal room is equipped with a high/low thermo-hygrometer and its own computerized controlled thermostat. Animal care staff monitor and record animal room high/low temperatures and humidity daily on the room activity log. Temperature settings are consistent with Guide recommendations and are calibrated by the Engineering Department. Alarm points are set at ± 4˚F. High or low temperature alarms are annunciated to the engineer on duty 24 hours a day. The Department of Animal Resources (DAR) management is notified of excursions. Most of the animal facilities are also equipped with an Edstrom Industries Watchdog environmental monitoring system in addition to the automated building management system (BMS). The Watchdog system registers temperature and humidity and also sends alarms to Animal Resources management personnel. Humidity levels are not controlled in any of the facilities but are reliably maintained between 30–70% most of the year.

Diet: Food (Teklad LM-485 autoclavable diet) is provided *ad libitum* to mice in wirebar lids. Water: our vivarium is equipped with a reverse osmosis (R/O) water purification system and automatic watering distribution system from Edstrom Industries. DAR receives monthly water quality reports from the City of San Diego. R/O purified water is monitored daily during the workweek. A number of parameters are monitored including conductivity, temperature, pH level and chlorine concentration. Automatic water delivery systems (room and rack distribution lines) are timed for daily in-line flushing. Quick disconnect drinking valves are sanitized with each cage change or more often if needed. System sanitation and preventive maintenance is performed by the DAR equipment technicians.

Acclimation period: mice are allowed up to 72 hours to stabilize into their new housing environment. Some experimental paradigms involve examining the behavioral response to novelty and therefore the animal cannot be habituated to the procedure.

Animal suffering: In order to minimize suffering, all surgical procedures are carried out under anesthesia using isoflurane (1–4%) in conjunction with ketamine/xylazine ip (90 120 mg/Kg and 10 mg/Kg). Mice are monitored every 15 minutes after induction for respiratory and heart rates if the surgical procedure requires more time. Animals are provided buprenorphine (0.05–2.5 mg/Kg s.c.) for 6–12 h followed by flunixine meglumine (2.5 mg/Kg s.c.) as a postoperative analgesic for 2 days post-implantation. Mice are observed 2 h, 6 h and 24 h post-surgery with daily monitoring during the course of the study. Mice are supplied with acidified water supplemented with sulfamethoxazole (or, sulfadiazine) with trimethoprim at a final concentration of 0.65–1.6 mg/mL to reduce chances of opportunistic bacterial colonization.

MUP-uPA-SCID/Beige mice were maintained at DAR at TSRI in accordance with protocols approved by the TSRI Ethics Committee, the Institutional Animal Care and Use Committee (Protocol Number: 11–0015). This study was carried out in strict accordance with the recommendations in the Guide for the Care and Use of Laboratory Animals of the National Institutes of Health. All efforts were made to minimize suffering. The method of sacrifice used for the experimental mice was cervical dislocation. A power calculation was used to determine the sample size (number of mice/group). 10 mice per group were used for each treatment in all experiments.

### HCV chimeric mouse study

Transgenic mice carrying the uPA gene driven by the major urinary protein promoter were crossed onto a SCID/Beige background (MUP-uPA-SCID/Beige) [31]. These transgenic mice are healthier than former Alb-uPA mice and provide an extended window from age 4 to 12 months for engraftment with human hepatocytes and infection with viral hepatitis viruses derived from serum of infected chimpanzees or from concentrated supernatant of HCV-replicating cell culture [31]. MUP-uPA-SCID/Beige mice (gift from A. Kumar) (4 months old) were transplanted with human hepatocytes (10^7^ cells/mouse) (we usually obtain ~300-500x10^6^ hepatocytes per donor, gift from D. Geller) as described previously [31]. In brief, fresh hepatocytes were transplanted immediately upon arrival within 12–16 hour after isolation. Viable cell counts were determined. One cm skin incision was made in the upper left quadrant of the mouse abdomen to visualize the spleen. Human hepatocytes were injected intra-splenically. Incision was then closed with Vetbond tissue adhesive (3M Animal Care Products, St. Paul, MN). To verify the degree of “humanization”, blood was weekly collected for human albumin quantification by ELISA (Bethyl Laboratories) according to the manufacturer’s protocol. Mice expressing >300 μg/mL of human albumin were randomized into groups of 10 mice. Note that the expression of the uPA transgene causes damage to the murine liver that is rapidly replaced by clusters of implanted human hepatocytes that can be visualized by human albumin immunostaining [31]. MUP-uPA-SCID/Beige mice that had been engrafted with human hepatocytes were infected intravenously (i.v.) with diluted plasma from HCV-infected chimpanzee (100 infectious doses (CID_50_)/mL of genotype 2a HCV) (gift from R. Lanford) or concentrated viruses (genotype 1a, 2a, 3a and 4a) derived from cell culture [16]. CRV431 was dissolved in polyethylene glycol 300 molecular weight (PEG-300), NV556 was dissolved in 5% absolute ethanol, 5% Cremophor EL, and 90% sterile saline solution, and sofosbuvir was dissolved in DMSO and subsequently in 95% sterile saline solution. Drugs were administered once by oral gavage at 50 mg/kg at the indicated time points. Blood was collected retro-orbitally at the indicated time points.

### Quantification of HCV RNA by Real-Time Reverse Transcription PCR

HCV RNA in serum and collected livers was extracted using the acid guanidinium-phenol-chloroform method. Quantification of HCV RNA was performed using real-time reverse transcription PCR based on TaqMan chemistry, as described previously [32].

## Results

### CRV431 and NV556 suppress ongoing HCV replication in humanized-liver chimeric mice

**Fig 1:**
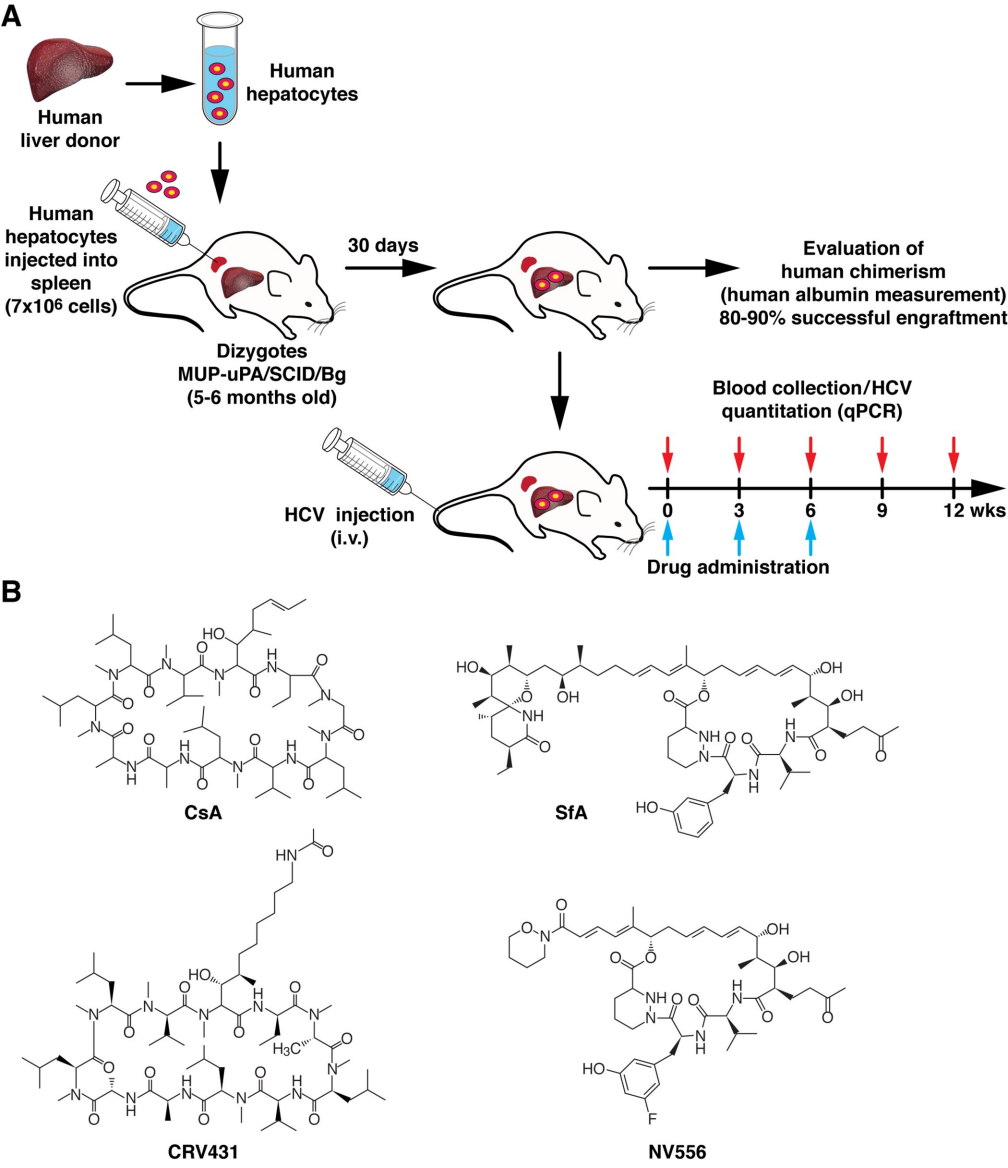
Experimental design for HCV infection of humanized-liver chimeric MUP-uPA-SCID/Beige mice. A. Experimental design for the creation, validation and role of humanized-liver chimeric mice. B. Chemical structures of cyclosporin A (CsA), the CsA analog CRV431, sanglifehrin A (SfA) and the SfA analog NV556.

We previously demonstrated and reported that two entirely structurally distinct non-immunosuppressive CypIs–the cyclosporin A analog CRV431 and the sanglifehrin analog NV556 (Fig 1)—efficiently inhibit HCV replication *in vitro* [13, 23]. The inhibition (nM range) was observed for all HCV genotypes tested [13, 23]. Here, we asked whether CRV431 and NV556 can also reduce HCV replication *in vivo*. To address this possibility, we used a chimeric mouse model, which consists of implanting permissive human hepatocytes into MUP-uPA-SCID/Beige mice (Fig 1) as developed and described by the Feinstone lab [31]. After verifying successful human hepatocyte engraftment by detecting high blood of human albumin (>300 ng/mL), MUP-uPA-SCID/Beige mice were infected intravenously with HCV. Viral replication was monitored over time by qPCR quantification and CRV431 and NV556 were administered orally at the indicated time points.

**Fig 2:**
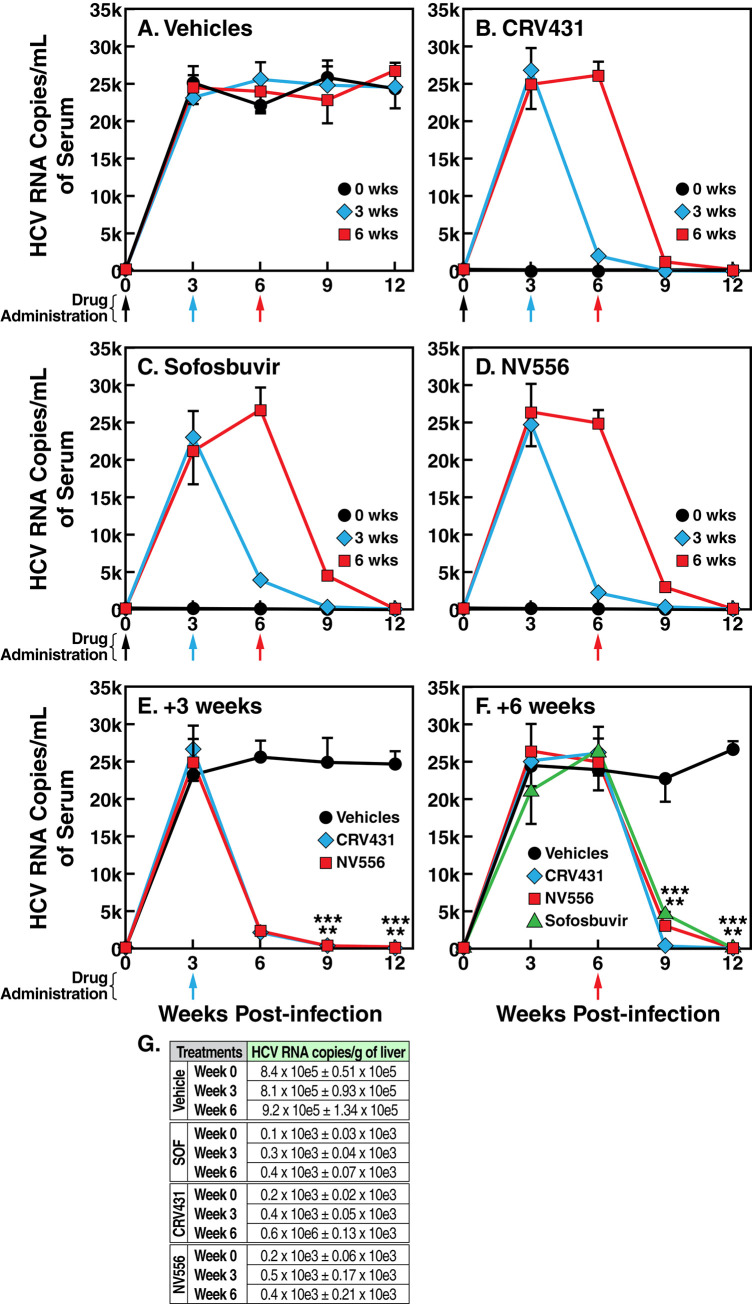
CRV431 and NV556 suppress HCV replication in humanized-liver chimeric mice. A-F. MUP-uPA-SCID-Beige mice implanted with fresh human hepatocytes were infected i.v. with diluted plasma from HCV-infected chimpanzee (100 infectious doses (CID_50_)/mL of genotype 2a HCV). Drugs (50 mg/kg) were administered orally at the indicated time points. CRV431 was dissolved in PEG-300, NV556 in 5% ethanol, 5% Cremophor EL, and 90% saline solution, and sofosbuvir in DMSO and subsequently in 95% saline solution. Blood was collected retro-orbitally every 3 weeks until week 12 post-HCV infection. For each treatment, 10 mice per group were used. Error bars represent standard deviation (SD) for the 10 mice per group. Statistical analysis was a protected ANOVA with Tukey-Kramer HSD Post-Hoc Test run in R 3.6.3. Asterisks indicate statistical significance (P less than 0.00001) in comparison to vehicles group at the same time point as determined by one-way ANOVA with post-hoc multiple comparisons tests (Tukey). G. Same as A except that livers were collected 12 weeks post-infection and HCV RNA levels were quantified as described previously [32]. Data (SD) are expressed as mean of HCV RNA copies per gram of liver collected from 10 mice per treatment.

In vehicle-treated chimeric mice, HCV replicates rapidly and reached a peak of viremia 9 weeks post-infection (Fig 2A). The administration of the NS5B polymerase sofosbuvir (50 mg/kg) together with HCV (T = 0) totally prevented viral replication in mice (Fig 2C). Similarly, the administration of either CRV431 or NV556 together with the virus completely abolished HCV infection (Fig 2B and 2D). Remarkably, CRV431 or NV556 administration (50 mg/kg) 3 or 6 weeks post-infection profoundly inhibited HCV replication 9 or 12 weeks post-infection (Fig 2E and 2F). To our knowledge, no previous studies investigated the inhibitory effect of CypIs in HCV-infected liver-humanized chimeric mice. Patterns of CRV431 and NV556-mediated viral inhibition were similar to that of sofosbuvir, indicating high potency of the two CypIs. CRV431- and NV556-mediated inhibitions of HCV RNA levels in livers (collected 12 weeks post-infection) correlate well with those of HCV RNA levels in serum (Fig 2G), likely reflecting a major neutralization of cyclophilin A in hepatocytes. Human albumin blood levels remained constant over the full period of drug treatments (12 weeks) and among all groups.

The CRV431- and NV556-mediated reduction of HCV replication is highly pronounced. For example, the administration of either CRV431 or NV556 6 weeks post-infection when viral replication is already robust (Fig 2F) suppresses viral loads 12 weeks post-infection (6 weeks post-drug administration) by >100-200-fold. Specifically, a reduction from 26,231 HCV RNA copies/mL in vehicle-treated mice to 112 and 221 copies/mL in CRV431- and NV556-treated mice, respectively. Evidently, CRV431- and NV556-mediated suppressions of viral replication 12 weeks post-infection (>100-200-fold or >99% decreases) are statistically significant (Fig 2E and 2F). Together these data indicate that the two structurally distinct CypIs—CRV431 and NV556—can totally suppress an established HCV infection in chimeric mice.

### CRV431 and NV556 suppress ongoing HCV replication in liver-humanized mice independently of the hepatocyte donor origin

**Fig 3:**
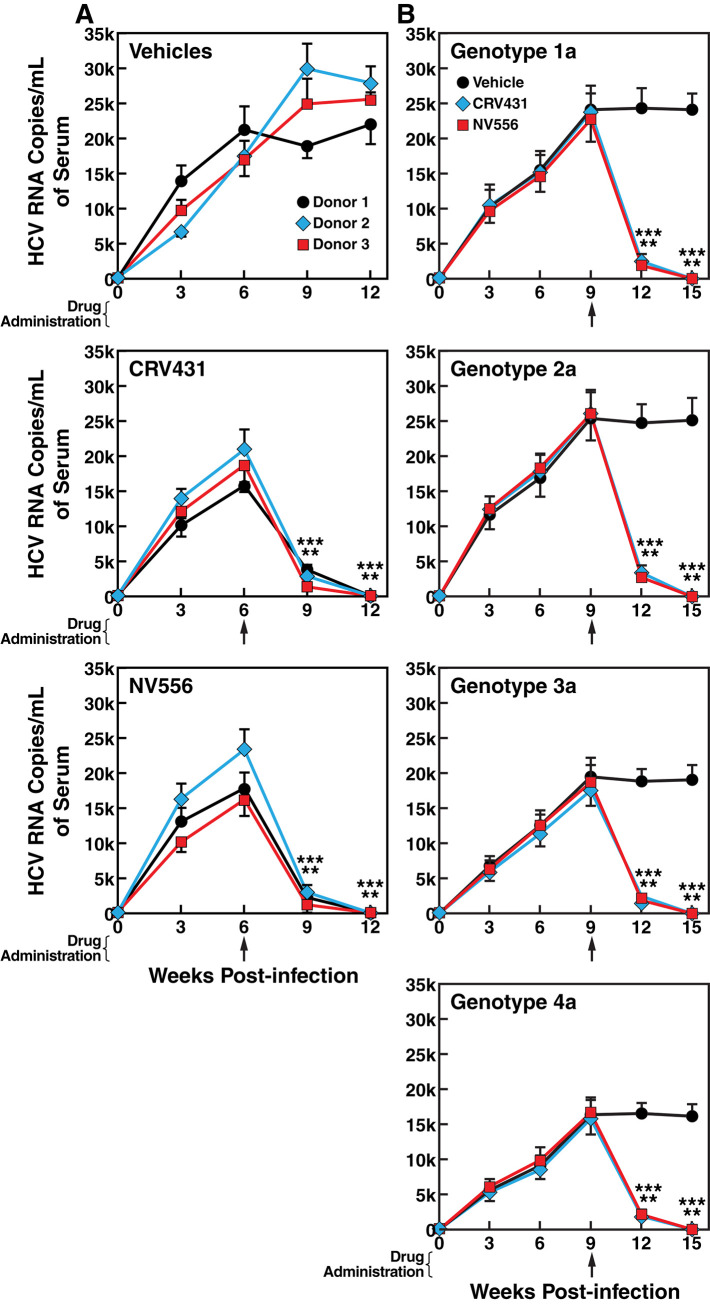
CRV431 and NV556 suppress HCV replication in chimeric mice independently of the donor origin of implanted human hepatocytes. A. MUP-uPA-SCID-Beige mice implanted with fresh human hepatocytes derived from three distinct donors were infected i.v. with diluted plasma from HCV-infected chimpanzee (100 infectious doses (CID_50_)/mL of genotype 2a HCV). Drugs (50 mg/kg) were administered orally 6 weeks post-infection. CRV431 was dissolved in PEG-300 and NV556 in 5% ethanol, 5% Cremophor EL. Blood was collected retro-orbitally every 3 weeks until week 12 post-HCV infection. B. Same as A except that chimeric mice were infected with concentrated HCV (100 infectious doses (CID_50_)/mL) from different GT (GT1a (H77), GT2a (J6/JFH-1), GT3a (S52) and GT4a (ED43)) [16] and drugs were administered 9 weeks post-infection. Blood was collected retro-orbitally every 3 weeks until week 15 post-infection. Data (SD) are expressed in HCV RNA copies per mL of serum. For each treatment, 10 mice per group were used (n = 10). Asterisks indicate statistical significance (P less than 0.00001) in comparison to vehicles group at the same time point as determined by one-way ANOVA with post-hoc multiple comparisons tests (Tukey).

We then asked whether the suppression of an established HCV infection can be repeated in mice implanted with human hepatocytes derived from several donors. To address this issue, human hepatocytes derived from 3 donors were implanted into MUP-uPA-SCID-Beige mice, which were then infected with HCV, and treated with vehicles, CRV431 and NV556 (50 mg/kg) six weeks post-infection. Blood levels of human albumin were similar between mice implanted with 7x10^6^ human hepatocytes derived from the three donors (315 +/- 17 ng/mL for donor 1, 352 +/- 62 ng/mL for donor 2, and 306 +/- 28 ng/mL for donor 3). Thirty mice per donor were randomized in 3 groups (vehicles, CRV431 and NV556 treatments (n = 10 per treatment). HCV replicated similarly in vehicles-treated chimeric mice implanted with hepatocytes derived from three donors (Fig 3A). Importantly, both the administration of CRV431 and NV556 6 weeks post-infection totally suppressed robust ongoing HCV replication 12 weeks post-infections (Fig 3A).

**Fig 4:**
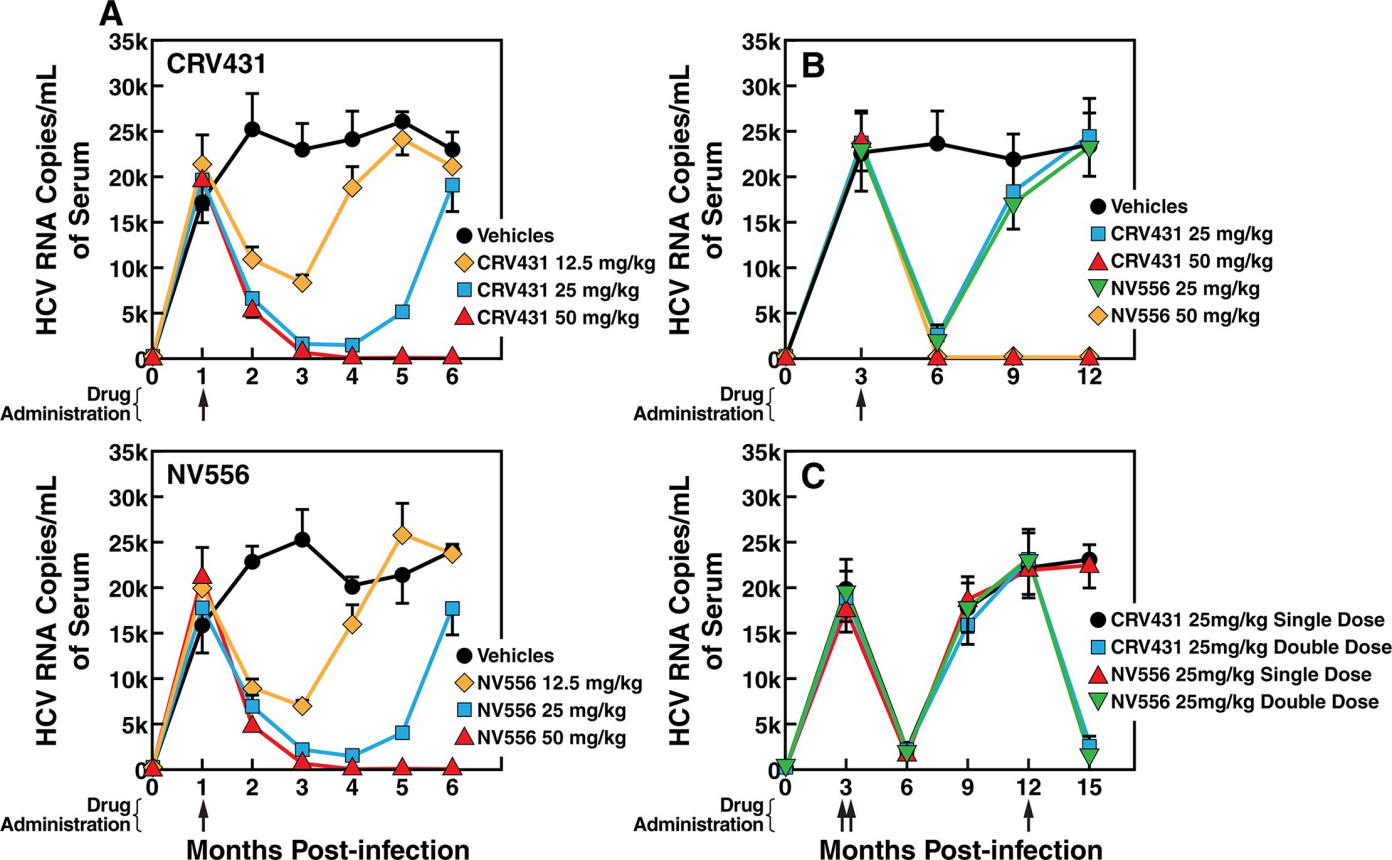
A single dose administration of CRV431 or NV556 prevents HCV rebound. A. MUP-uPA-SCID-Beige mice implanted with fresh human hepatocytes were infected i.v. with diluted plasma from HCV-infected chimpanzee (100 infectious doses (CID_50_)/mL of genotype 2a HCV). CRV431 or NV556 (12.5, 25 or 50 mg/kg) were administered orally 4 weeks (1 month)-post infection. CRV431 was dissolved in PEG-300 and NV556 in 5% ethanol, 5% Cremophor EL. Blood was collected retro-orbitally every month until month 6 post-HCV infection. B. Same as A except that drugs were administered 3 months post-infection. C. Same as A except single low drug dose was administered 3 months post-infection while double low drug doses were administered 3 and 9 months post-infection. For each treatment, 10 mice per group were used (n = 10). The X-axis shows the “months post-infection” rather than “weeks post-infection”.

The CRV431- and NV556-mediated inhibitions of HCV replication remain highly pronounced among hepatocyte donors. For example, the administration of either CRV431 or NV556 6 weeks post-infection when viral replication is already robust (Fig 3A) reduces viral loads 12 weeks post-infection (6 weeks post-drug administration) by >150-250-fold. For donor 1, a reduction from 22,118 HCV RNA copies/mL in vehicle-treated mice to 85 and 133 copies/mL in CRV431- and NV556-treated mice, respectively. For donor 2, a reduction from 26,998 HCV RNA copies/mL in vehicle-treated mice to 106 and 159 copies/mL in CRV431- and NV556-treated mice, respectively. For donor 3, a reduction from 25,094 HCV RNA copies/mL in vehicle-treated mice to 97 and 126 copies/mL in CRV431- and NV556-treated mice, respectively. We obtained similar profiles of a suppression of viral replications when CRV431 and NV556 were administered 9 or even 12 weeks post-infections (Figs 3B and 4B, respectively). These data demonstrate that the two totally unrelated structurally CypIs—CRV431 and NV556—suppress ongoing HCV replication in mice with liver reconstituted with human hepatocytes derived three donors. We observed a similar inhibition of viral replication for different HCV genotypes (GT1a, GT2a, GT3a and GT4a) when CRV431 and NV556 were administered at the peak of viral replication (9 weeks post-infection) (Fig 3B). This confirms previous studies including ours demonstrating that CypIs—CRV431, NV556 and alisporivir—are pan-genotypic [12–13, 16–19, 23].

### Prevention of HCV rebound by CRV431 and NV556 is dose-dependent

We then asked whether CRV431 and NV556 can prevent viral rebound after single dose drug administration. Specifically, HCV-infected chimeric mice received 4 weeks post-infection a single dose of 12.5, 25 and 50 mg/kg of CRV431 or NV556, and viral replication was monitored monthly over a period of 6 months. We prolonged the period of viral load analysis from 3 months (12 weeks) (Figs 2 and 3) to 6 (Fig 4A), 12 (Fig 4B) and even 15 months (Fig 4C) to permit an eventual occurrence of a viral rebound after an extended period of time after CypI treatment in complementary experimental designs. Therefore, data in Fig 4 are expressed in “months post-infection” rather than “weeks post-infection”. In vehicle-treated chimeric mice, viral replication was relatively stable 2 to 6 months post-infection (Fig 4A). A single 12.5 mg/kg dose of CRV431 or NV556 4 weeks post-infection partially reduced HCV replication 3 months post-drug administration, but viral replication rebound after 5 months (Fig 4A). A single 25 mg/kg dose of CRV431 or NV556 4 weeks post-infection profoundly suppressed HCV replication 3 months post-drug administration, but viral replication nevertheless rebound after 6 months (Fig 4A). In contrast, a single 50 mg/kg dose of CRV431 or NV556 4 weeks post-infection totally suppressed reduced HCV replication 4 months post-drug administration, and most importantly, no viral rebound was observed after 6 months (Fig 4A). These data suggest that a single 50 mg/kg dose suppresses HCV infection in the absence of viral rebound when added 1 month post-infection.

We conducted a similar experiment but this time CRV431 and NV556 were added 3 months post-infection when viral replication is maximal and steady (Fig 4B). A single low dose of CRV431 or NV556 (25 mg/kg) profoundly reduces viral replication 3 months post-drug administration (6 months post-infection), but again viral rebound occurs 6 months post-drug administration (9 months post-infection) (Fig 4B). In contrast, a single high dose of CRV431 or NV556 (50 mg/kg) suppresses viral replication and apparently prevents viral rebound even 9 months post-drug administration (12 months post-infection) (Fig 4B). To exclude the possibility that the viral rebound observed after a single low dose of CRV431 or NV556 (25 mg/kg) (Fig 4B) results from CRV431- and NV556-resistant variants, a second low dose of CRV431 or NV556 (25 mg/kg) was administered when viral rebound is maximal (9 months post-single dose administration or 12 months post-infection) (Fig 4C). The second low dose of CRV431 and NV556 for a second time profoundly reduces viral replication, suggesting that the first viral rebound unlikely originated from CRV431- or NV556-resistant escape variants but rather from the residual pool of wild-type viruses. This is in accordance with previous studies including ours [16–19] that CypI present a high barrier to the development of viral resistance.

## Discussion

Although a majority of DAAs-treated patients achieve virological cure, HCV resistance to DAAs has a crucial role in the failure of pIFNa-free treatment regimens. The presence of viral variants resistant to NS5A inhibitors at baseline is associated with lower rates of HCV cure in certain groups of patients, such as those with genotype 1a or 3, those with cirrhosis, and/or prior non-responders to pIFNa-based regimens [33]. DAA-resistant HCV variants represent the main cause of virological failure. Viruses resistant to protease inhibitors disappear from blood in a few weeks to months, while NS5A inhibitor-resistant viruses remain for years [33]. Re-treatment options are available, but first-line treatment strategies should be optimized to efficiently prevent treatment failure due to HCV resistance [33]. The failure of one regimen does not imply that the patient cannot be cured from HCV infection since another scheme is always applied, and if it fails (which is very rare), a third one can be used. So according to our current knowledge, the existence of multiple regimens allows clearance of infection from any patient. One of these regimens may include an additional class of drugs with high barrier resistance and with mechanisms of antiviral action distinct from current DAAs. We previously demonstrated that the *in vitro* combination of CypI with NS5A inhibitors provides additive to synergistic anti-HCV activity without detectable cross-resistance [16]. The new *in vivo* data presented in this study suggest that such a class of drug candidates are the CypIs. Specifically, we showed that a single administration of CRV431 or NV556 (50 mg/kg) when viral replication is robust and well established in humanized-liver MUP-uPA-SCID-Beige mice drastically suppressed viral replication. We obtained similar results for HCV genotype 1a, 2a, 3a and 4a. Importantly, a single high dose of CRV431 or NV556 (50 mg/kg) prevents viral rebound even after 9 months post-single dose CypI administration, suggesting a high barrier to the development of viral resistance. Supporting this hypothesis, we found that when viral rebound occurs when using a single low dose of CypI (25 mg/kg), the virus remains sensitive to a second low dose of CypI. Thus, these data together with previous work [16–19] indicate that CypI display a high barrier to the development of viral resistance.

CRV431 and NV556 exhibit apparent low toxicity *in vivo*. Indeed, we found that a daily oral administration of a high dose of CRV431 or NV556 (50 mg/kg) for extended periods of time (i.e., 14 to 30 weeks treatment) in mice did not induce unanticipated side effects [34–36]. This is in accordance with previous work conducted in humans that showed that a daily administration of the CypI alisporivir alone or in combination with ribavirin in HCV-infected patients in phase I, II, and III studies did not induce significant adverse effects [16–22].

Our recent work that show that both CRV431 and NV556 significantly decrease the development of liver fibrosis and hepatocellular carcinoma in non-alcoholic steatohepatitis mouse models [34–36], our present data suggest that the two entirely structurally different CypIs—CRV431 and NV556—derived from unrelated natural products, represent attractive partners to current DAA regimens for the treatment of hepatitis C and liver diseases.
